# Population estimation and harm reduction among people who inject drugs in Addis Ababa, Ethiopia

**DOI:** 10.1186/s12954-020-00407-x

**Published:** 2020-09-07

**Authors:** Negussie Deyessa, Bekele Senbete, Aman Abdo, Bernard M. Mundia

**Affiliations:** 1 Department of Preventive Medicine, School of Public Health, College of Health Sciences, Addis Ababa University, P.O. Box 3253, Addis Ababa, Ethiopia; 2 Organization for Social Services, Health, and Development, Addis Ababa, Ethiopia; 3 Kenya AIDS Non-Governments Consortium (KANCO), Nairobi, Kenya

**Keywords:** Population estimate, People-who-inject-drugs, Harm reduction, HIV/AIDS, Ethiopia

## Abstract

**Background:**

Injecting drug use is known to contribute significantly to the spread of the HIV epidemic in many parts of the developing world. Due to the hidden nature and stigma of the problem, it is difficult to study using routine surveys. Therefore, this study aims to estimate the number of people who inject drugs in Addis Ababa, Ethiopia, and to describe the epidemiological and social situation related to HIV among people who inject drugs.

**Methods:**

The study used rapid assessment methods, followed by combined methods of estimating populations, using nomination and multiplier methods. The combined methods used two datasets: the first includes the proportion of people who use services within a year as a multiplier, and the second, a count of the list of people with a problem who used the specific service within a year as a benchmark. The rapid assessment incorporated different qualitative tools to elicit information related to injectable drugs, using existing data sources, in-depth interviews, and focus group discussions.

**Results:**

The study estimated a total of 4068; with 95% CI (3196, 5207) people who inject drugs (PWIDs) in Addis Ababa. The study found people who inject drugs were young in age, male, with a lower educational status, unmarried, and living in small clerical business. People who inject drugs and participated in the study were more likely to use additional substances like alcohol, khat, and cannabis. The most common form of injectable drug used was heroin, and most of the people who inject drugs reported sharing syringes and needles. A high proportion of study subjects also disclosed having positive test results for HIV, hepatitis B, and C.

**Conclusion:**

The population size of people who inject drugs in Addis Ababa is high. Lack of service in harm reduction in the city has made PWIDs vulnerable and at higher risk for HIV/AIDs and hepatitis B and C. Therefore, responsible bodies must start implementing the essential harm reduction strategies given by the World Health Organization.

## Background

Injecting drug use is known to contribute significantly to the spread of the HIV epidemic in many parts of the developed and developing world [1–3]. The sharing of injection needles among drug users is the major route for HIV transmission [4–6]. Of the blood-borne infectious agents, HIV contributes substantially to the high morbidity and mortality caused by illicit drug use [7, 8]. HIV prevalence among people who inject drugs is much higher than the general public [2, 3]. Injecting drug use is also considered the primary mode of transmission of HIV in the world, excluding in Sub-Saharan countries. In many resource-limited countries, including Ethiopia, injecting drug use is becoming the second most common route for transmitting HIV [8].

HIV risk behavior is higher among women than among men who inject drugs [9, 10]. In a study by Magnus et al., in 2013 in Washington, DC, women who inject drugs reported higher HIV risk behaviors than men who inject drugs [9]. HIV prevalence is also higher among sex workers who inject drugs than sex workers who do not inject drugs [11, 12].

A high proportion of people who use injection drugs live also with HIV [1, 2, 13], hepatitis C [13–19], and hepatitis B [13, 18, 20]. People who inject drug are often marginalized and are socially stigmatized and discriminated within health and social care settings [21–23]. People who inject drugs are prone to problems difficult to accept in a legal environment. They are involved in different forms of abuse, considered as criminals that are easily arrested [24, 25]. People who inject drugs have no friendly healthcare services and make them more vulnerable to share unclean needles [26]. In many countries, utilization of the services of people who inject drugs is falling below the lower target outlined by the WHO [26, 27].

People who inject drugs, like other substance users, are at higher risk for serious morbidity and mortality due to an overdose of the drug [28, 29]. Studies have shown that heroin overdose is a significant cause of mortality for injection drug users [30, 31]. Injecting drug users are also more prone to common mental disorders than the general public [32].

Research on people who inject drugs is hindered by methods of estimating the size of a hidden population. Literature shows the lack of a single method which could be described as the ‘gold standard’ technique for population size estimation. The methods used for the estimation of the population of hidden phenomena include the census/enumeration method, simple population survey method, the nomination method, and multiplier methods. Each of these methods has their own advantages and limitations [33, 34]. Combining the different estimation methods is a useful strategy as it allows for triangulation and contextualization of the finding [35].

Research on injection drug use is also challenging due to lack of information on the number of affected people and geographic location over time. Although the existence of people who inject drugs in major cities and towns in Ethiopia is well known, its occurrence and the estimated number are not well documented. Such information could be used to better target HIV and hepatitis C prevention programs among people who inject drugs and to compare the occurrence of injection drug use, HIV, and hepatitis C across the cities [36–39]. These data are also important for policy-makers, service providers, and government health authorities to offer appropriate services. Knowledge of the number of injection drug users within a population would aid both health authorities and community organizations in assessing the coverage of existing programs and in the planning and delivery of a range of public health services. Therefore, the aim of this research was to estimate the number of people who inject drugs in Addis Ababa and to describe the epidemiological and social situation related to HIV among people who inject drugs.

## Methods

### Design and setting

The study used a rapid assessment method, followed by a combined method, using the nomination and multiplier method. The rapid assessment incorporated several components to elicit information related to hotspot sites of injectable drug use within the city. This included in-depth interviews with experts, people who inject drugs and key informants as well as focus group discussions, site mapping, simple observations, and a review of relevant documents.

The research was conducted in Addis Ababa, the capital city of Ethiopia, which also serves as the headquarters location for many national and international organizations, including the African Union. It is a center for more than 120 embassies and a place of residence for many tourists. The city has a total population of 5.65 million (projected for 2017), with an average density of 5646 people per square kilometer [40].

### Study population

The source population for the research included people who inject drugs and others who are linked with this group. To estimate the number of people who inject drugs, the source population were people who inject drugs, with the following inclusion criteria: at least 18 years of age, who use an injection drug for non-medical purposes, who stayed in the study city for at least the last 6 months, and who used an injection drug at least once in the last 3 months. Cases who fulfilled the above criteria and who were captured by the respondent-driven sampling method were assessed for injection drugs use with a confirmatory checklist. The confirmatory checklist included (1) knowledge of injection sites on the body; (2) knowledge of where to obtain injection rigs; (3) knowledge of the size of syringes and needle and on how to use it; and (4) assessing scar at the point of the last injection on the body. Eligibility was assessed by a trained coordinator. People who inject drugs were sampled through a respondent-driven sampling (RDS) approach for face-to-face interviews until saturation was reached. Respondent-driven sampling is a variant of chain referral sampling and was used to recruit peers. When implemented and analyzed properly, RDS can provide estimates representative of the networks of the populations sampled [41, 42]. The source population for the rapid situational assessment included people who inject drugs, syringe providers, people working to reduce the problem, health providers, local leaders, religious leaders, commercial sex workers, truck drivers, leaders of networks and organizations, the police, relevant staff from institutions of higher learning, and governmental and non-governmental organizations (NGOs) working with people who inject drugs.

### Sampling and recruitment

**Fig. 1:**
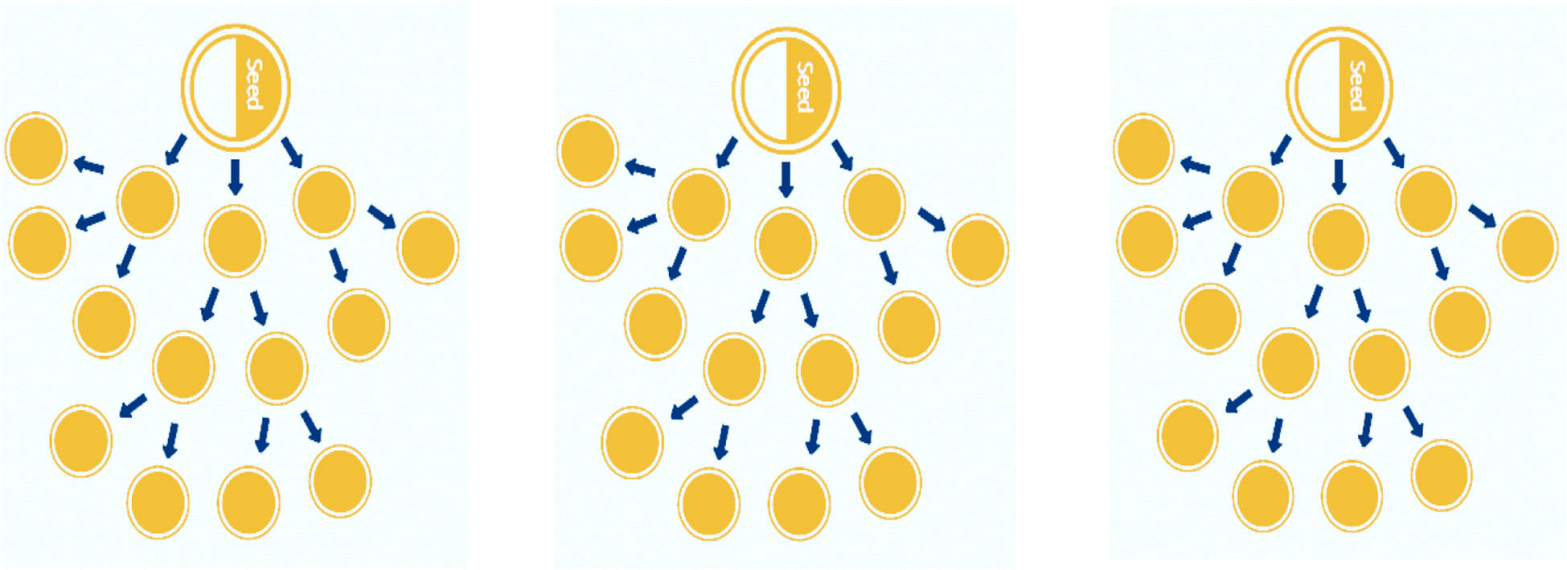
Scheme of respondent-driven sampling

Since the study estimated the total population who inject drugs, sample size determination was not considered. However, maximizing inclusion of people who inject drugs was necessary. In the combined methods, for both the nomination and multiplier methods, all people who inject drugs and captured by the respondent-driven sampling in the given time period were included. For the qualitative methods, data collection continued until saturation of information related to the research questions was reached. In each enumeration site, three individuals who inject drugs who were selected by the rapid assessment were selected as seeds for the respondent-driven sampling approach. Emphasis was placed on ensuring diversity among the individuals selected s seeds. Each seed was oriented individually on the objectives of the study, and the use of coupons to recruit three eligible participants. After enrolling and completing the behavioral questions, each seed was given a fixed number (three) of coupons o recruit other peers with the same behavior of the seed as part of the first wave of recruitment. The second wave of recruitment included people who inject drugs, who came with a coupon of recruitment provided by recruits included in the first wave of recruitment (Fig. 1).

Each recruit was interviewed for the estimation, on questions related to behaviors and distribution of the problem in PWIDs. All recruits were subsequently advised to recruit another maximum of three peers who inject drugs. The successive trend of recruitment was ensured in long recruitment chains of people who inject drugs, and it continued until it was difficult to find any additional recruits of PWIDs. The recruitment process of study subjects was monitored through the unique number-coded coupons provided on each participant’s recruitment coupon.

### Data collection process

For the primary objective, to estimate the population size and prevalence of people who inject drugs, using the nomination method, participants were further interviewed through asking two questions, broadly of the following sort: “How many friends of your own, who inject drugs regularly in the last year, do you know?” and “How many of these you know have received a treatment service, due to problems related to the drug, in the last one year in a government hospital in the city?” From these two answers, the multiplier was estimated for health facilities as benchmarks. The multiplier was multiplied by total PWIDs in the selected health facilities who used the service in the last year as a benchmark.

To find the benchmark for the city, the research group assessed the number of people who used treatment facilities due to problems related to their use of drugs within a year of the current data collection. Using data extraction from the logbooks of seven hospitals in Addis Ababa, the total number of PWIDs residing in the city was extracted to use as a benchmark. Multiplying the two sets provides an estimate of the probable size of the population in the community. During data collection, descriptive information was collected from study subjects found during the assessment.

Within each enumeration site, two enumerators with previous data collection experience conducted face-to-face interviews. A questionnaire with unique identifiers for each respondent, socio-demographic characteristics, type of drugs injected (including frequency and recency), and geographical and social locations of injecting drug users (village level) was developed. The data collectors were trained on safety and how to approach people who inject drugs. Because of its sensitive nature, the training included information on counseling services, providing a coupon for cases, safety, and ethical issues in addition to the content of the questionnaire. Great caution was made to avoid repeated data collection of eligible study subjects. The questionnaire was translated into the local language and piloted in a similar setting. Participants were reimbursed for their transportation to take part in the study and for bringing three PWIDs.

For the rapid situational assessment, interview guide and the checklist were developed to guide the data collection process. The data collection was led by the principal investigator and a note taker, and interviews were recorded using an audio recorder. The interview guide was developed based on the main themes of the study and included questions on the social, legal and policy environment shaping patterns of injection drug use, and linked health risks; the extent and nature of access to care and HIV prevention services among people who inject drugs; and the HIV prevention and harm reduction interventions in need of development and their likely possibility. The data collector was free to explore emergent issues in the field while using the interview guides. All qualitative interviews were assisted by the rapporteur and some were audio-recorded with the consent of participants. In addition, a document review was conducted in close consultation with the facility staff.

### Data analysis

Data collected for estimation of the number of people who inject drugs were entered into a computer using EPI-DATA version 3.0 software package with a double-entry scheme using a programmed entry template, and validated on a daily basis. Descriptive analysis was conducted to assess demographic characteristics and assess reported problems encountered among people who inject drugs. For the estimation of people who inject drugs, both the multiplier and nomination methods used data from two independent sources to examine the overlap between the two sources. The first source was a count of PWIDs from abstracted logbook treated in the last year from seven hospitals in Addis Ababa, as a benchmark, and the second source was the proportion of PWIDs who were considered to use the above hospitals in Addis Ababa, in the last year.

To estimate the total number of people who inject drugs using either nomination or multiplier methods, the total number of PWIDs using the seven hospitals was divided by the multiplier. The total number of PWIDs using each hospital, although called the benchmark is similar for each method, the difference is in the estimation of the multiplier. Furthermore, the 95% CI of the multiplier was made by taking the 95% confidence level and considering the multiplier and the total sample.

$$ \mathrm{Total}\ \mathrm{PWIDs}=\frac{\mathrm{Total}\ \mathrm{participants}\ \mathrm{on}\ \mathrm{PWID}}{\mathrm{PWIDs}\ \mathrm{using}\ \mathrm{hospitals}\ \mathrm{within}\ \mathrm{a}\ \mathrm{year}}\left|\mathrm{x}\ \mathrm{PWID}\mathrm{s}\ \mathrm{using}\ \mathrm{the}\ \mathrm{hospitals}\right. $$

The following formula was used to estimate the multiplier for the nomination method:

$$ \mathrm{Total}\ \mathrm{PWIDs}=\frac{{\mathrm{M}}_1+{\mathrm{M}}_2+{\mathrm{M}}_3+{\mathrm{M}}_4+\dots \dots {\mathrm{M}}_{\mathrm{n}}}{{\mathrm{C}}_1+{\mathrm{C}}_2+{\mathrm{C}}_3+{\mathrm{C}}_4+\dots \dots {\mathrm{C}}_{\mathrm{n}}}\mathrm{x}\ \mathrm{PWIDs}\ \mathrm{using}\ \mathrm{the}\ \mathrm{hospitals} $$

Where C1, C2, C3, …. Cn was the number of nominee PWIDs who use a specific the seven hospitals, within a year time, by each individual participating PWID in the survey; whereas M1, M2, M3,…. Mn was the number of nominee PWIDs known by each individual who participated in the survey. The calculation for the multiplier method was the proportion of PWIDs in the survey who claimed to use service provided in the hospitals within a year as the multiplier for the benchmark. To synthesize a single estimate, the multipliers were pooled estimates, calculated with the fixed model effect, giving weight to the size of people in the survey [multiplier method] and size of a total number of nominees PWIDs known by participants in the survey. The benchmark was calculated by summing the total count from each hospital. The total population estimate was calculated using a simple excel calculator in Microsoft Office. The single estimate was further supported by its 95% confidence limit.

The qualitative interviews, for the rapid situational assessment, were transcribed verbatim and then translated into English by the qualitative interviewers. The English version was prepared in text files and entered into epi.dat computer software to handle qualitative data. Data analysis was made by grouping into the main themes of the study in Microsoft Excel. Based on the deep understanding of the context and the findings from the quantitative study, the qualitative findings were used to complement the quantitative finding and for triangulation of the findings.

## Results

The study included 276 people who inject drugs. Almost three-quarters of study subjects were below 35 years of age, with a mean age of 29.8 years and SD of + 8.1 years, ranging between 18 and 67 years. The majority, almost 9 of the 10 study subjects, were males, and two-fifths had not completed elementary classes. About half of the study subjects reported any form of employment. Clerical jobs were most common, but a substantial proportion of study subjects were students in high school and universities (Table 1).

**Table 1:** Socio-demographic characteristics of people who inject drugs in Addis Ababa and Hawassa cities, December 2019

Characteristics	Frequency	Percent
Age group		
Less than 25	69	25.0
25–34	136	49.3
35–44	54	19.3
45+	17	6.2
Mean + SD	30.5 + 8.1	
Range	20 to 67	
Sex		
Male	260	94.2
Female	16	5.8
Educational status		
Elementary	113	40.9
Secondary	95	34.4
Tertiary	68	24.6
Marital status		
Single	229	83.0
Married	24	8.7
Divo/Wid/Sepa	23	8.3
Religion		
Orthodox	230	83.3
Muslim	26	9.4
Protestant	14	5.1
Others	6	2.2
Occupation		
Students	64	23.2
Employed(gov/NGO)	12	4.3
Self-employed	87	31.5
Small clerk business/trade	113	40.9
Monthly income (*n* = 240)		
< 1000 Birr	102	42.5
1000–1900 Birr	65	27.1
2000 Birr or more	73	30.4
Mean + SD (Birr)	1664 + 2348 Birr	
Enumeration		
Zewditu (site 2)	137	49.6
OSSHD (site 1)	139	49.6

Almost 70% of the PWIDs reported drinking alcohol daily, 60% reported chewing khat, and 60% reported consuming hard non-injection substances on a daily basis. However, only a few study subjects reported not drinking alcohol, chewing khating, or consuming any non-injectable hard substances in the last 12 months (Table 2).

**Table 2:** The pattern of people who inject drugs on the use of other substances in Addis Ababa and Hawassa, December 2019

Substance	Frequency	Percent
Alcohol		
Daily	191	69.2
1–2 times a week	17	6.2
Rarely	39	14.1
Never drink	29	10.5
Khat		
Daily	162	58.7
1–2 times a week	28	10.1
Rarely	45	16.3
Never chew	41	14.9
Hard (non-injectable) substance		
Daily	163	59.1
1–2 times a week	56	20.3
Rarely	43	15.6
Never drink	14	5.1

Almost half of the study subjects consumed injectable drugs for the first time during their teenage years, while more than nine out of 10 study subjects reported starting to take injectable drugs when they were below 30 years. More than a quarter of the study subjects reported the last injection was within 24 h to a week of the interview, while one in three PWIDs were using the drug 2–3 times per day, and one in nine were using it on a weekly to monthly basis (Table 3).

**Table 3:** Characteristics of drugs used for injection and process of its use by people who inject drugs substances in Addis Ababa and Hawassa, December 2019

Characteristics	Frequency	Percent
Age at first injection		
Below 20 years	130	47.1
20–29 years	119	43.1
30 years or more	27	9.8
Mean + SD	22.1 + 6.1	
The last injection		
Today/yesterday	45	16.3
Within a week	51	18.5
Within a month	44	15.9
Frequency of injection		
Daily to 2–3 per day	29	33.0
Once in a week to a month	168	60.9
Less frequent (> 3 months)	17	6.2

Study subjects reported using several types of drugs. The most commonly used drugs were heroin, followed by cocaine and pethidine Almost one in four reported taking two or more types of drugs. The drugs were reportedly obtained from friends, special shops, smuggled from contrabands, from pharmacies or drug shops, or stolen from governmental and non-governmental health facilities. The average daily cost per drug and cost per single injection episode was less expensive to a statistically significant level for study subjects from Hawassa compared to subjects from Addis Ababa (Table 4).

**Table 4:** Characteristics of commonly used drugs in the past 12 months by people who inject drugs substances in Addis Ababa and Hawassa, December 2019

Characteristics of drugs	Frequency	Percent
Drug type		
Heroin	155	56.2
Cocaine	133	48.2
Pethidine	43	15.6
Crack	11	4.0
Morphine	6	2.2
Ecstasy	3	1.1
Tramadol	2	0.7
Others	4	1.4
Number of type of drugs		
Only one	209	75.7
Two drugs	58	21.0
Three or more	9	3.3
Place where a drug is found		
From friends	227	82.2
Special shops	43	15.6
Smuggled from contraband	28	10.1
From pharmacy/drug shops	22	8.0
Non-gover. health facility	17	6.2
From an ordinary shop	7	2.7
Reported cost of drug	Ethiopian Birr (Mean + SD)	
Average daily price	189.39 + 158.1	
Price of drugs used on the last day	172.77 + 149.0	
Price for a single injection	145.96 + 127.9	
Price of a single injection of the last time	144.70 + 129.1	

The study estimated the population size of people who inject drugs using nomination and multiplier methods. The benchmark of drug users who attended hospitals in Addis Ababa was obtained from six hospitals. A total of 1044 people who inject drugs were found under treatment for a problem due to injectable substance use, in the year 2017. In this study, only 74.2% of the PWIDs interviewed had reported visiting a health facilities once in the last 12 months. Considering the captured PWIDs represent the PWID population, the total benchmark for the Addis Ababa will be 775 PWIDs (Table 6).

Based on the survey, using the nomination method, the multiplier for attending health facilities in Addis Ababa was 5.2747, while for imprisonment it was 5.5207. Based on the nomination for using health facilities, the study estimated a total number of 4088; 95% CI, (3758, 4481) people who inject drugs. Synthesized estimation was calculated by taking a pooled estimate using a fixed-effects model, and a total of 4068 (95% CI; (3196, 5207) adult people who inject drugs were available in Addis Ababa (Table 5).

**Table 5:** Number of PWIDs and their friends who visited health facility due to their drug use habit, for population estimation using nomination/multipliers, in Addis Ababa, December 2019

Types of PWID	Number	Multiplier	Benchmark	Estimated population	(95% CI)
Notification method					
Number of friends of the participants	2131				
Number of friends attending HF	404	5.2747	775	4088	(3758, 4481)
Multiplier method					
Number of PWIDs contacted	276				
Number of PWIDs using HF	54	5.11111	775	3961	(3196; 5207)
Overall estimate		5.248517	775	4068	(3196, 5207)

The majority of study subjects from Addis Ababa (72.5%) reported reusing needles. Substantially, a higher proportion (18%) of study subjects claimed to reuse syringe and needle, once, twice, or more times. Moreover, study subjects disclosed sharing needles with other people, in some cases up to four or more people and some of which were unknown to the study subjects (Table 6).

**Table 6:** Characteristics and pattern of using syringe and needle by people who inject drugs in Addis Ababa and Hawassa, December 2019

Syringe and needle use pattern	Frequency	Percent
Use of new syringe and needle for the last injection		
Yes	76	27.5
No	200	72.5
Frequency of using previously used syringe/needle		
Never shared	225	81.5
One or two times	29	10.5
Three or more times	22	8.0
Number of people who used a syringe in common		
One to 3 other people	18	35.3
Four or more people	33	64.7
Number of new people who shared a syringe		
No one	14	27.5
One to two	33	64.7
Three or more people	4	7.8

People who inject drugs were asked whether they had been tested for certain diseases. Of the 177 who reported having been tested for HIV, 70 (39.5%) disclosed as having HIV-positive results. Moreover, of the 99 people who reported having been tested for hepatitis B, 37 (37.4%) reported having the disease. Of the 46 who reported having been tested for hepatitis C, 13 (28.3%) reported testing positive for the disease. Of the 171 who reported being tested for other sexually transmitted diseases, 66 (38.6%) disclosed receiving a positive test (Table 7).

**Table 7:** Diseases related to the behavior of people who inject drugs, [only Addis Ababa data] in Addis Ababa, December 2019

Disease entity (reported diagnosis)	Sample (tested)	Number (positives)	Percent
HIV test	177	70	39.5
Hepatitis B test	99	37	37.4
Hepatitis C test	46	13	28.3
Other STD (history last 12 months)	171	66	38.6

## Discussion

The study, the first to estimate injection drug use in a metropolitan city in Ethiopia, estimated a total of 4068 (95% CI; (3196, 5207) people who inject drugs in Addis Ababa, Ethiopia. As this is a hidden population, and there are methodological challenges to estimating the magnitude of people who inject drugs, our findings may represent an overestimation or underestimation. Although we had planned to collect data from health providers and police detention registers as a benchmark, obtaining information only from health providers may have affected our estimates and limited potential comparisons. However, our estimation methodology used the nomination and multiplier methods and generated a pooled measure of the two estimates and their confidence limits in order to obtain a proxy estimation of PWIDs in the city. However, in the measurement of the pooled estimate, a fixed-effects model of pooling the multiplier, and providing a narrow confidence limit may underestimate the number of people who inject the drug in the city. The other problem that may not be corrected here was the difficulty of representation of PWIDs from the rich community who may not be assessed by respondent-driven sampling with such relatively low incentive.

The study found people who inject drugs to be young in age, male, with low educational status, unmarried, and working as clerks in small businesses. People who inject drugs who participated in the study were more likely to use additional substances such as alcohol, khat, and cannabis and were using the substances frequently.

As young people are prone to risk-taking behaviors, and cities in developing countries have a high proportion of young people, it was expected that youth would comprise much of our sample. Furthermore, many studies also support that young people are prone to substance use [43–45]. Similarly, research from other contexts also reports a higher proportion of men compared to women who use injection drugs [46–48].

Our study has also reports people who inject drugs in Addis Ababa to have a lower educational level and work in a small clerk business; although the study population did include people with all levels of education. This may be due to the fact that there is a proportion of uneducated people in the cities, as evident in the census [26, 46]. Other studies suggest that people with a lower level of education may be more likely to initiate injection drug use that could result in addiction [46]. The high proportion of unmarried PWIDs in this study may represent difficulty in finding a partner and to marry when having an addiction or alternatively some respondents may have been divorced or may not have disclosed their marital status as it is stigmatizing [49].

Many study subjects started injection drugs at a young age and reported frequent use at least once per week. Initiation of drug use at an early age may be related to the fact that many youth engage in high-risk acts when they are encouraged by their peers. Injection drug use initiated among the youth has been reported in other studies [47, 48].

The most common form of the drug used in the city was heroin, and the dominance of one or two types of drugs in the community may be due to the clustering effect of living in common localities. Although the majority of the PWIDs inject a single form of the drug, some PWIDs report taking two or more drugs. The major source of drugs among the PWIDs was obtaining drugs from friends. The hidden nature of the drug use may have made users depend only on a few people for their supply. PWIDs also repored that drugs could be found in special shops or it may be found after it is smuggled. These drugs are considered to be expensive, and drug use could disrupt family life, with some PWIDs having to leave their job, to sell their cars and houses and to divorce or get separated from their marriage and leave their family dispersed in streets [50, 51].

In the quantitative data, only a few study subjects disclosed sharing needles; however, the qualitative data suggested a higher level of needle sharing. Needle sharing is a common phenomenon among PWIDs and increases risk of HIV transmission. People who inject drugs usually buy and use drugs communally. Such use of drugs in groups is important to minimize the costs of the drugs and needles and syringes. PWIDs also disclosed taking an injection in common as a sign of sensing a commonness and friendship.

In this study, data from Addis Ababa showed high reports of positive tests for HIV, hepatitis B, and C. Since people who inject drugs report risky behaviors such as needle sharing, the prevalence of these diseases may be higher than the general population. The high prevalence could also relate to lower comprehensive knowledge of transmission and prevention of HIV among people who inject drugs. It should be noted that our study presents perceived diagnoses, which may not be accurate. Finally, the study found the absence of major services related to harm reduction among people who inject drugs in Ethiopia. The stigmatizing and discriminating nature of injecting drug use, augmented by the criminalization of the drug, has resulted in difficulty in reaching people who inject drugs, and in limited access of PWIDs to appropriate services.

## Conclusion

The study estimated a total of 4068 (95% CI (3758, 5207) people who inject drugs in Addis Ababa, Ethiopia. This large estimated population size is of relevance to policy-makers in major cities where large numbers of PWIDs may also be present but hidden. In Addis Ababa, the youth are the most vulnerable population for injection drug use, thus government should assess and initiate strong prevention strategies within instituitions where youth may be found, such as schools [elementary, secondary, and tertiary level]. The most common type of injection drug used in this study was heroin, which can be partially treated by opioid replacement therapy. Such replacement therapy should be made available in the city. A high proportion of study subjects reported having a positive test result for HIV, and hepatitis B and C. However, these results needs confirmation using biological sero-testing method for HIV and viral hepatitis, and indivuals should be linked with the appropriate treatment and care HIV and viral hepatitis.

## Data Availability

Data and materials are fully available through requesting ND.

## Abbreviations

AIDS: Acquired immunodeficiency syndrome
HIV: Human immunodeficiency virus
PWID: People who inject drugs
